# Calculus as a Risk Factor for Periodontal Disease: Narrative Review on Treatment Indications When the Response to Scaling and Root Planing Is Inadequate

**DOI:** 10.3390/dj10100195

**Published:** 2022-10-20

**Authors:** Stephen K. Harrel, Charles M. Cobb, Lee N. Sheldon, Michael P. Rethman, John S. Sottosanti

**Affiliations:** 1 Department of Periodontics, Texas A&M College of Dentistry, 3302 Gaston Avenue, Dallas, TX 75246, USA; 2 Department of Periodontics, School of Dentistry, University of Missouri-Kansas City, 650 East 25th Street, Kansas City, MO 64108, USA; 3 Private Practice, 2223 Sarno Road, Melbourne, FL 32935, USA; 4 School of Dental Medicine, University of Maryland, Baltimore, MD 21201, USA; 5 College of Dentistry Columbus, The Ohio State University, OH 1233 S. Lakeview Drive, Prescott, AZ 86301, USA; 6 Private Practice, 8899 University Center Lane, Suite 100, San Diego, CA 92122, USA

**Keywords:** periodontitis, dental calculus, dental scaling, root planing, biofilms, inflammation

## Abstract

Background: Based on the 2018 classification of periodontal disease, a series of articles have been published describing the decision points of periodontal therapy and how the findings collected at those decision points can be used as guidelines for periodontal therapy. The articles are reviewed with a focus on the finding of inadequate calculus removal at the decision points and how that finding impacts treatment outcomes. Methods: A narrative review of the literature discussing the influence of calculus on inflammation was performed and the effects of inadequate removal of calculus during periodontal therapy were analyzed in light of the 2018 classification of periodontal disease, the decision points of periodontal therapy, and the guidelines of periodontal therapy. Results: The published literature supports that calculus is a major risk factor for periodontal inflammation. Recent studies indicate that the pathologic risk of calculus goes beyond the retention of biofilm and may represent a different pathophysiologic pathway for periodontal disease separate from the direct action of biofilm. The inadequate removal of calculus is a factor in the failure of periodontal therapy. Conclusions: The inadequate removal of calculus plays an important role in the frequent failure of non-surgical periodontal therapy to eliminate inflammation.

## 1. Introduction

In 2018 the American Academy of Periodontology (AAP) and the European Federation of Periodontists (EFP) concurrently published a new classification of periodontal disease [1,2]. The classification divided periodontal disease into four Stages, starting from the earliest presentation of periodontitis moving to the most advanced presentation. Further, this classification of periodontal disease was divided into three Grades that designated the progression rate of disease with Grade A being slow progression through Grade C being rapid progression. The 2018 classification has become the standard classification for the periodontal diseases. Using this classification, it is possible to assign a level of disease at all patient evaluations (Stage 1–4) and, after two or more evaluations, assign a rate (Grade A–C) of disease progression. Table 1 summarizes the AAP/EFP classification parameters.

While the new classification gives definitive parameters for the stage and grade of periodontal disease, it does not give treatment recommendations for the different stages. Treatment was addressed by the EFP in 2020 [3]. While multiple treatment possibilities were outlined by the EFP, specific decision points to guide clinical treatment were difficult to ascertain. Due to the need for clinical guidance, specific decision points for treatment were developed by a group of academic and clinical periodontists with many years of experience in the private practice of periodontics, teaching of periodontics within an academic institution, and clinical periodontal research. These decision points were outlined by Harrel et al. in 2022 [4]. The decision points were based on the 2018 classification system, and they defined points during the clinical treatment of a patient with periodontal disease where reassessment of the patient’s periodontal condition was required. The purpose of the decision points was to define where reevaluation of the response to the treatment performed up to that point should be assessed so a determination could be made as to whether treatment had been successful. Based on the finding of the reevaluation, a decision could then be made regarding whether the patient could be placed in a periodontal maintenance program or if more advanced periodontal therapy was indicated. The decision points aided in defining critical phases where further evaluation was necessary but did not define what further treatment may be indicated. Later in 2022, the same group who had developed the decision points published clinical guidelines for periodontal therapy [5,6]. These guidelines for periodontal treatment combined the 2018 classification of periodontal disease with the previously defined decision points in therapy.

All clinical treatment of periodontal disease is based on the basic tenet of eliminating inflammation and then maintaining an inflammation free state [7,8,9]. At a purely scientific level this requires the removal of any contaminated material from the periodontal sulcus and the establishment of a stable microbiota of non-harmful organisms. The clinical application of this goal is stated simply as the removal of plaque and calculus from the tooth and establishing acceptable conditions for the patient to perform adequate oral hygiene. All clinicians who treat periodontitis know that accomplishing these goals can be very difficult. If these goals are not achieved, there will always be a continuation of periodontal inflammation and an ongoing risk for progression of periodontitis. The determination of whether these goals have been accomplished and maintained can only be achieved by routine and rigorous reevaluations of the patient’s periodontal condition.

If signs of inflammation are present at the reevaluation following initial periodontal treatment or at any periodontal maintenance appointment, further periodontal treatment is indicated. This may be a repetition of the treatment performed during initial therapy, but in most instances advanced periodontal therapy is required. Advanced periodontal therapy may take the form of non-surgical debridement with advanced visualization, localized minimally invasive surgery, or generalized surgical access. In all instances, whether non-surgical or surgical, advanced therapy starts with gaining visual access to accomplish instrumentation for the removal of calculus that was not removed during initial therapy. After calculus removal, advanced therapy may also include surgical treatment to allow for improved oral hygiene (osseous surgery) or regeneration of the damaged periodontal supporting structure. However, the common denominator needed for the success of advanced therapy is the removal of calculus that remains after initial therapy. Thus, the need for advanced therapy is based on a therapist’s inability to remove calculus during initial therapy. The retention of calculus is central to the instances where there is a failure of debridement procedures and the progression of periodontal disease following treatment [10].

This narrative review will look at the following clinically relevant issues: (1) The previously published decision points and guidelines as they apply to clinical decisions for further periodontal treatment; (2) The role of calculus and the incomplete removal of calculus during initial therapy on the need for advanced periodontal treatment; (3) The pathologic risk factors for periodontal disease that can be addressed by the practitioner performing active therapy; and (4) The ethical considerations of periodontal reevaluations and the recommendation for further therapy.

## 2. Initial Therapy

In all cases, no matter what stage of the disease, the initial treatment of periodontitis will consist of debridement of the teeth and the periodontal sulcus in conjunction with oral hygiene instructions (OHI). This debridement generally takes the form of scaling and root planing (SRP) [10]. In most instances, SRP is performed simultaneously with OHI aimed at teaching the patient how to remove the constantly renewed plaque and biofilm on their teeth. While it is well recognized that OHI is an integral part of maintaining periodontal health, the actual application of the OHI and the daily maintenance of oral hygiene is in the hands of the patient [11]. Because this is a patient dependent activity, this portion of periodontal therapy will not be addressed in this paper other than to state that oral hygiene is mandatory for clinical success and should be evaluated, reviewed, and reinforced at every appointment.

## 3. Debridement and the Removal of Calculus

**Figure 1:**
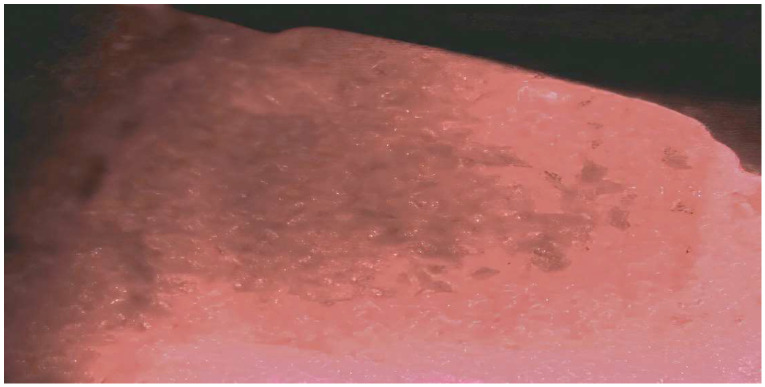
Microislands of calculus remaining on a root surface after ultrasonic and hand scaling until the root of the extracted tooth was deemed calculus free when visualized with 3.5× magnification loupes. The root surface is illuminated with a 655 nm laser to aid visualization of calculus. (Videoscope photo 40× magnification).

SRP is the most frequently performed periodontal treatment procedure [12]. Part of the definition of SRP is the complete removal of all soft and hard deposits on the tooth and root [13]. The required complete removal of hard material (calculus) from the root surface is technically and physically very demanding and becomes more difficult as the periodontal sulcus becomes deeper with periodontal disease progression [14]. Several studies have looked at the efficacy of performing SRP without enhanced (endoscope or videoscope) visualization [15,16,17,18]. SRP without enhanced visualization is often referred to as “blind SRP”. These studies found that when an expert clinician with adequate instrumentation and time performed blind SRP, between 27 to 73% of the planed root surface still had detectable calculus following subsequent surgical exposure of the root surface [19]. Studies have also shown that when extracted teeth that had SRP performed using direct visualization under 3.5× loupe magnification with unfettered access for instrumentation, 20% of the root surface continued to have what were termed microislands of calculus remaining [20]. Figure 1 shows microislands of calculus remaining after SRP using loupes for magnification. A recent scanning electron microscope (SEM) study has shown that residual calculus fragments, referred to as fractured calculus, are present on root surfaces that have undergone SRP [10]. It is likely that the fractured calculus seen with SEM may represent the microislands of calculus detected in other studies. These studies collectively reveal that a residuum of subgingival calculus remains in most instances where SRP has been performed in a routine “blind” manner. This routine retention of calculus is a major factor in disease recurrence and represents incomplete and inadequate treatment for the control of periodontal disease [10].

## 4. Calculus as a Risk Factor for Periodontal Degeneration

There is general agreement that plaque/biofilm consisting of a community of bacteria and other microbiota, e.g., viruses and protozoa, is the initial cause of periodontal disease [21,22,23,24,25,26,27]. Because of the extensive literature supporting plaque as the “cause” of periodontal disease, there has been a relative deemphasis on the role of calculus in the persistence and progression of periodontal disease. Most have indicated that calculus plays a secondary role acting mainly in the retention of plaque and that the removal of calculus is solely to allow for improved oral hygiene [28].

However, there is both classic and recent literature indicating that calculus plays a more direct role in periodontal inflammation and destruction. For example, an in vivo animal study showed that sterile calculus when placed in Guinea pigs resulted in generalized inflammation and granulation tissue production [29]. In addition, a study using non-surgical endoscopic visualization in humans revealed that nearly 70% of inflammation (assessed via tissue coloration) of the soft tissue wall in deep periodontal pockets was associated with calculus covered by biofilm and less than 20% of the inflammation was associated with biofilm alone [30].

Recently a potential alternate pathway for cell death in periodontal tissues has been reported. Ziauddin et al. [31]. demonstrated that sterile calculus, when phagocytized by connective tissue cells in cell culture, induced cell death [31]. In a later study, Ziauddin et al. [32]. confirmed the cytotoxic effects of sterile calculus using an in vitro model consisting of HSC-2 oral epithelial cells and THP-1 macrophages. This pathway of cell death was described as pyroptosis, i.e., an inflammatory form of lytic programmed cell death. The authors postulated that the calculus crystals are phagocytized by the epithelial cells utilizing a defense mechanism designed to bring bacterial products into the cell where they are neutralized. However, when the phagocytized agent is the crystallin structure of calculus, the cells die. While this pathway of induced cell death has yet to be demonstrated in humans, it is logical to presume that such a pathway will act in humans in a similar fashion as seen in the cell culture studies [33]. If this presumption were to prove true, then it explains the clinical observations that most inflammation in the gingival sulcus soft tissue is associated with calculus and that residual calculus is found at sites of inflammation following SRP.

## 5. Reevaluation after Scaling and Root Planing

As previously mentioned, the initial treatment of periodontal disease routinely consists of OHI and debridement of the tooth by SRP. This treatment is designed to reduce inflammation of the periodontium. It is clinically and ethically mandatory that a thorough reevaluation be performed after this initial treatment to determine if the therapeutic goal of inflammation control has been achieved. The control of inflammation is usually determined by the absence of bleeding on probing (BOP) and the reduction of pocket probing depths to an acceptable level [12]. This returns us to the decision points and clinical guidelines discussed earlier [5,6]. Ideally, at the post SRP decision point, all patients would be returned to periodontal health. Regrettably, this goal is frequently not achieved.

Unfortunately, the result of initial SRP and OHI is often only an improvement of the periodontal condition but not an elimination of inflammation and the disease process. This is often clinically evident by patients continuing to have BOP at reevaluation or a return of BOP at a maintenance appointment. Based on the use of the AAP/EFP classification, if the patient has a continuation or return of inflammation, they have active periodontal disease. In the presence of ongoing active periodontal disease, the patient should not be placed in a maintenance program but should be treated with some form of advanced periodontal therapy. Based on the recently published decision points and guidelines, it is rare that repeated SRP will materially correct the residual or recurrent inflammation. The repeated use of a non-successful SRP treatment is not clinically or ethically acceptable.

**Figure 2:**
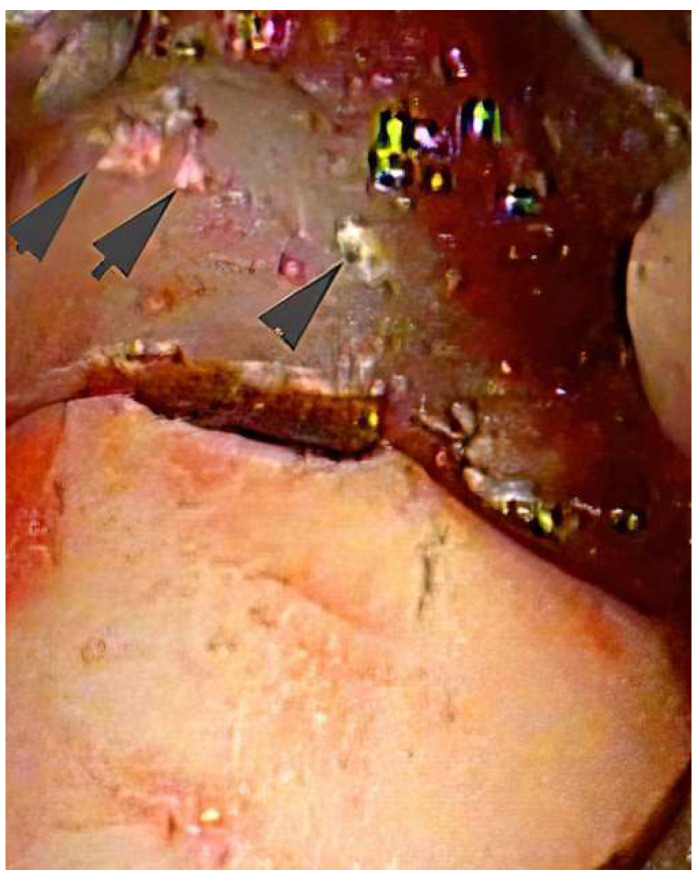
During videoscope assisted minimally invasive surgery (VMIS), multiple areas of residual calculus (arrows) are noted to remain after SRP. The position of the calculus is in an area of deep pocket probing depth and would be very difficult to access during blind SRP. (Videoscope photo 40× magnification).

**Figure 3:**
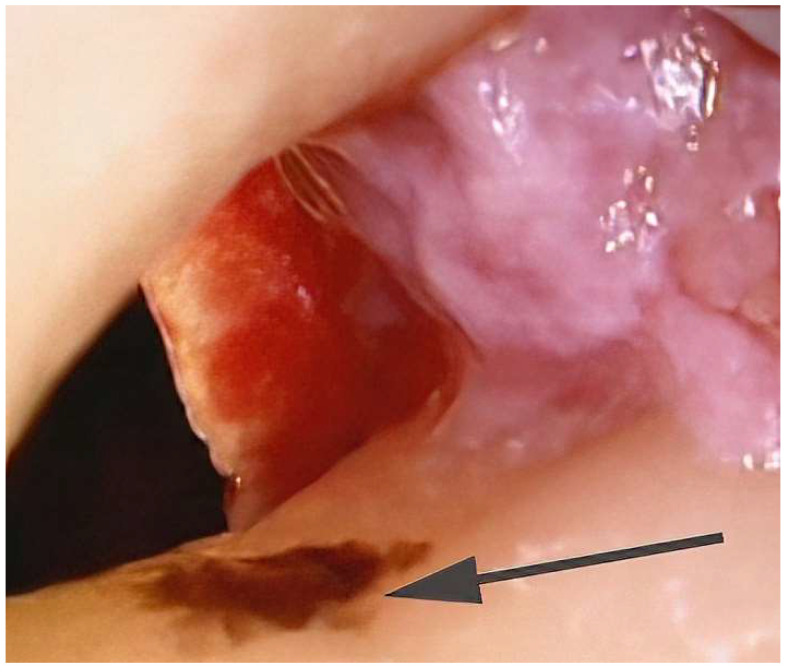
During osseous surgery an area of calculus (arrow) missed during SRP is shown in a concavity on the root surface. Due to the anatomy of the root, this calculus would be very difficult to remove without direct visualization. (Videoscope photo 40× magnification).

In most cases where SRP is not successful in controlling inflammation, clinical experience indicates that there is usually subgingival calculus present that has not been removed. Figure 2 and Figure 3 show areas of residual subgingival calculus remaining after SRP that was performed on an anesthetized patient by a periodontist. Because most sulcular inflammation is associated with calculus, it is necessary to remove the residual calculus with advanced therapy [10,30]. Advanced therapy can range from the use of enhanced non-surgical visualization with an endoscope or videoscope to surgical access. Both the use of enhanced visualization or surgical access are aimed at improved visualization for the removal of residual calculus. The surgical removal of calculus may be an initial step in a procedure aimed at regeneration of lost periodontal tissues. However, for a successful regenerative procedure it is necessary to have a calculus free root surface to prevent a return of inflammation [20,30]. The studies showing that calculus may represent an alternate pathway from plaque that leads to epithelial cell death gives further weight to the necessity of removing all calculus [31,32,33]. The use of chelating agents for root modification and the removal of microislands of calculus may be a further necessary step beyond current routine surgical and non-surgical methods [20].

## 6. Treatment Recommendation Based on Reevaluation of Therapy

After initial debridement (SRP) and OHI, the patient should be reevaluated to determine their response to therapy. The timing of this reevaluation can be from 6 weeks to 3 months post-initial therapy depending on case type. More complex and advanced cases (Stage 3 to 4) should be reevaluated sooner because they will almost always require advanced periodontal therapy. If the patient’s inflammation is under control at reevaluation with no BOP and the pocket probing depths are at a maintainable level (usually ≤ 4 mm), it is reasonable to place the patient on a maintenance schedule at 3 to 6 month intervals. Inflammation should be reevaluated at each maintenance interval. If the patient continues to have inflammation, evidenced by BOP and/or deep pocket probing depths that are not maintainable by the patient with oral hygiene, advanced therapy aimed at removing residual calculus and possible regeneration should be recommended for the patient. The exact type of advanced therapy will vary with the patient, the characteristics of their condition, and the skill level of the practitioner.

It is ethically unacceptable to place the patient on a maintenance schedule if they continue to have active disease. Active disease requires active treatment. It is also ethically unacceptable to continue to treat the patient with inadequate therapy such as repeated SRP that does not yield improved results. The patient must be informed of the lack of satisfactory results and the availability of advanced therapy with the discussion recorded in the patient’s records. In all cases, any advanced therapy must be performed at the level that would be delivered by a specialist. If the needed advanced therapy is beyond the skill level of the treating practitioner, the patient should be referred for specialty care [5].

Occasionally the patient may choose to not pursue advanced therapy due to insurance coverage, national healthcare coverage, and personal or other reasons. While this presents a difficulty for the treating practitioner, it does not excuse the necessity of informing the patient of the availability of advanced therapy and that without such therapy their periodontal disease will likely progress and lead to loss of teeth. Patients also should be informed that a referral to a periodontist may be appropriate. This scenario also raises questions regarding the role of chronic periodontal inflammation as a risk factor in various systemic diseases, e.g., atherosclerosis, cardiovascular disease, ischemic stroke, Alzheimer’s disease, etc. [34,35,36,37,38]. The patient must be informed of the potential systemic risks of inadequate periodontal treatment that allows for continued inflammation.

This narrative review is aimed at informing the reader of recent publications establishing clinical decision points and guidelines for periodontal therapy. It is not designed to be a definitive literature review such as would be presented in a systematic review. For further information, the reader is urged to review the AAP/AFP classification and the papers previously published by the authors of this paper on decision points and guidelines for periodontal therapy [1,2,3,4,5,6]. The patient is dependent on the practitioner to inform them of their health or lack of health. If the response to a performed treatment has been inadequate to render periodontal health, it is ethically mandatory that the patient be informed that the treatment results have not been completely successful, and that further treatment is available. Not informing the patient of continued inflammation and disease progression is ethically unacceptable.

## Figures and Tables

**Table 1:** A simplified diagnostic and treatment summary drawn from the staging criteria from the 2018 Classification of Periodontal and Peri-implant Diseases and Conditions. The original classification papers should be consulted for details.

Periodontal Disease Stage	Pocket Probing Depth	Interproximal Attachment Loss (CAL)	Type of Bone Loss	Percent Bone Loss	Number of Teeth Lost	Other Factors	Probable Treatment Needs
Stage I	4 mm or less	1 to 2 mm	Mostly Horizontal	Up to 15%	None	None	SRP
Stage II	5 mm or less	4 to 5 mm	Mostly Horizontal	15–33%	None	Minimal	SRP and/or Advanced
Stage III	6 mm or Greater	5 mm or Greater	Vertical and Horizontal	More than 33%	4 or Less	Many	SRP and Advanced
Stage IV	6 mm or Greater	5 mm or Greater	Vertical and Horizontal	More than 33%	5 or more	Many and Complex	SRP and Advanced

## Data Availability

Not applicable.
